# Dasatinib + Gefitinib, a non platinum-based combination with enhanced growth inhibitory, anti-migratory and anti-invasive potency against human ovarian cancer cells

**DOI:** 10.1186/s13048-017-0319-2

**Published:** 2017-04-26

**Authors:** Benoît Thibault, Bertrand Jean-Claude

**Affiliations:** 1 Research Institute – McGill University Health Center (MUHC), 1001 Décarie Blvd, Block E, Montreal, QC H4A 3J1 Canada; 2 Present Address: INSERM – Cancer Research Center of Toulouse (CRCT), 2 avenue Hubert Curien, Toulouse, France

**Keywords:** Ovarian cancer, EGFR, c-Src, Met, Kinase inhibitors, Signaling pathways, Dasatinib, Gefitinib, Equieffective treatment

## Abstract

**Background:**

Ovarian cancer is the leading cause of death for gynecological cancers and the 6th cause of women cancer death in developed countries. The late stage detection, the peritoneal dissemination and the acquisition of resistance against carboplatin are the main reasons to explain this poor prognosis and strengthen the need of alternative treatments to improve the management of ovarian cancer and/or to sensitize tumors to platinum salts. Epidermal growth factor receptor (EGFR), hepatocyte growth factor receptor (Met) and cellular Src kinase (c-Src) are crucial kinases implied in ovarian tumor growth, survival, invasion and resistance to carboplatin. Their expression is increased in advanced ovarian cancers and is correlated with poor prognosis. Despite a clear potential in inhibiting these proteins in ovarian cancer, as a single agent or in combination with a carboplatin treatment, we need to target kinases in tandem because of their capacity to trigger compensatory pathways that synergize to promote drug resistance.

**Methods:**

Here we target EGFR, c-Src and Met individually or in combination with carboplatin, using Gefitinib, Dasatinib and Crizotinib respectively, in a panel of carboplatin-sensitive (OVCAR-3, IGROV-1 and A2780) and carboplatin-resistant cells (SKOV-3 and EFO-21). We studied the ability of the most potent combination to induce apoptosis, regulate migration, invasion and to modulate the activation of proliferation and survival proteins.

**Results:**

Crizotinib, Dasatinib and Gefitinib, alone or in combination with carboplatin, showed a cell-specific cytotoxic synergy in ovarian cancer cells. The Dasatinib plus Gefitinib combination was synergistic in OVCAR-3, SKOV-3 and, in IGROV-1 cells (high concentrations). This combination was unable to induce apoptosis but suppressed cell migration, invasion and the activation of EGFR, Erk, c-Src and Akt compared to single treatments.

**Conclusions:**

Combining carboplatin with kinase inhibitors lead to synergistic interactions in a cell-specific manner. Unlike platinum-based combinations, mixing Dasatinib with Gefitinib led to cytotoxic activity, inhibition of cell migration and invasion. Thus, the Dasatinib + Gefitinib combination presents anti-tumour properties that are superior to those of platinum-based combinations, indicating that it may well represent a promising new treatment modality to be tested in the clinic.

**Electronic supplementary material:**

The online version of this article (doi:10.1186/s13048-017-0319-2) contains supplementary material, which is available to authorized users.

## Background

Ovarian cancer is the leading cause of death for gynecological cancers and constitutes the 6th cause of women cancer death in developed countries [1]. This high mortality is mainly due to a late diagnosis (at stage III and IV for 70% of patients), a peritoneal dissemination of cancer cells and an innate or acquired resistance to chemotherapy. Indeed, despite the fact most patients respond to the initial carboplatin and taxanes reference treatment (70 to 80%), recurrence frequently occurs with the acquisition of a resistance to carboplatin [2, 3]. Thus, new treatment modalities are urgently needed to sensitize patients to carboplatin thereby improving the management of ovarian cancer.

Ovarian tumors are often characterized by an overexpression and activation of tyrosine kinase receptors that are implied in tumor progression and resistance to carboplatin [4–11]. Among these proteins, the epidermal growth factor receptor (EGFR), hepatocyte growth factor receptor (Met) and cellular Src kinase (c-Src) are key regulators of tumor cell proliferation, survival and invasion [12–15]. EGFR is overexpressed in most of ovarian carcinomas compared to normal tissues and has been associated with poor prognosis [4–6]. However, despite a clear implication of EGFR in cancer progression, clinical results with EGFR inhibitors were disappointing in ovarian cancer. As an example, the inhibition of this receptor by Gefitinib in a phase II clinical trial was well tolerated but showed minimal anti-tumor activity [16]. Encouraging results showing anti-tumor efficacy when the inhibition of EGFR is combined with the targeting of phosphatidylinositol 3-kinase (PI3K) indicate the necessity to inhibit alternative pathways triggered by EGFR inactivation [17].

c-Src is an intracellular member of the Src kinase family that activates, among others, EGFR or the MAP kinase pathway via Ras [18, 19]. The inactivation of c-Src by an antisense strategy induces tumor and vascular network growth inhibition in a murine model of SKOV-3 ovarian cancer cell xenograft [20]. Another example showed that the inhibition of c-Src with AP23994 leads to the inhibition of tumor growth and angiogenesis in mice models of ovarian tumors with a maximum effect in combination with docetaxel [21]. c-Src is overexpressed and activated in late-stage ovarian cancers but not in normal ovarian epithelium [7]. Numerous human ovarian adenocarcinoma cells (HOAC) are sensitive to Dasatinib, an inhibitor of c-Src, which synergizes with carboplatin and paclitaxel in a cell-specific manner [8, 9]. A phase II clinical trial in platinum-resistant ovarian cancer patients showed no advantage in the association of Saracatinib, a dual inhibitor of Src and Abl, with paclitaxel [22]. Dasatinib, which entered in phase I in ovarian cancer patients, could however show more promising results because of its action on Src, Bcl-Abl, c-kit, PDGFβ and other kinases [23].

Met, whose natural ligand is Hepatocyte Growth Factor (HGF), can recruit various adapter proteins or direct kinase substrates through its Src-Homoloy-2 (SH2) domain such as PI3K, Src or Ras and is implied in tumor growth, survival, invasion and in matrix remodeling [13]. The Met overexpression in advanced epithelial ovarian cancer is a marker of poor prognosis and its inhibition has been showed to be critical to prevent tumor progression [24–26]. Indeed, the inhibition of this protein by PF-2341066 in a mouse model of ovarian cancer diminishes tumor growth and increases animal survival [27]. Likewise, Foretinib, an inhibitor of Met and VEGFR-2, is responsible for an inhibition of tumor growth and metastasis in a mouse model of ovarian cancer, mainly due to apoptosis activation and a reduction of tumor cell adhesion, migration, invasion and proliferation [28]. Another inhibitor of Met, MK8033, sensitizes OVCA cells to carboplatin and paclitaxel, confirming the need of targeting the Met pathway in this pathology [10].

Despite a clear implication of EGFR, c-Src and Met in ovarian cancer progression and resistance to carboplatin, the inhibition of these proteins alone could be futile because of their capacity to stimulate the resistance between each other and the synergies they are able to establish. Indeed, in head and neck cancer, c-Src is able to stimulate Met which induces resistance to erlotinib, an EGFR inhibitor [29]. Moreover, in tumor cells, Met is able to bind EGFR and to be phosphorylated without an upstream HGF activation [30]. On the contrary, the HGF-mediated Met activation triggers resistance to Gefitinib, an inhibitor of EGFR in lung adenocarcinoma cells [31]. Numerous synergies established between EGFR and c-Src and responsible for an increased tumor growth have also been described, strengthening the necessity to target these two proteins in tandem [32].

Here, we decided to inhibit individually or in tandem EGFR, c-Src and Met using Gefitinib, Dasatinib and Crizotinib respectively, the latter being pharmacological inhibitors commonly used in clinic. We associated these treatments with carboplatin to determine a potential capacity of kinase inhibitors, alone or in combination, to sensitize HOAC to platinum salts. We evaluated the cytotoxic activity of all these treatments on a panel of platinum-sensitive (OVCAR-3, IGROV-1 and A2780) or platinum-resistant (SKOV-3 and EFO-21) cell lines. Because the Gefitinib and Dasatinib combination showed a potent cytotoxic synergy in some of our HOAC, we decided to further study the mechanism of action underlining this effect. We focused our work on the capacity of the Gefitinib and Dasatinib combination to induce apoptosis and to regulate both migration and invasion. We finally studied the effects of this combination on HOAC cell proliferation and survival signaling pathways, in particular EGFR, Erk, c-Src, and Akt.

## Methods

### Drugs

Carboplatin was provided by the Royal Victoria Hospital pharmacy at a concentration of 26.9 mM in saline solution. Crizotinib was purchased from PharmaBlock (USA), Inc. (CA, USA). Gefitinib and Dasatinib were purchased from the Royal Victoria Hospital (Montreal, Canada) pharmacy and extracted from pills in our laboratory. All kinase inhibitors were dissolved in DMSO to obtain a concentration of 20 mM maximum. Drug dilutions were carried out under sterile conditions using RPMI (10% FBS) medium and the final concentration of DMSO never exceeded 1% (v/v).

### Cell culture

Human ovarian adenocarcinoma cell (HOAC) lines NIH-OVCAR-3 and SKOV-3 (ATCC® numbers HTB-161 and HTB-77) were obtained from the American Type Culture Collection (Manassas, VA). HOAC line IGROV-1 was a gift from the Gustave Roussy Institute, Villejuif. A2780 cells were obtained from Sigma Aldrich (France). EFO-21 cell line was obtained from the Deutsche Sammlung von Mikroorganismen und Zellkulturen GmbH (DSMZ, Germany). HOAC were cultured in RPMI 1640 medium containing 10% of fetal bovine serum, supplemented with with 10% FBS, 10 mM HEPES, 2 mM L-glutamine, gentamycin sulfate and fungizone (all reagents purchased from Wisent Inc., St-Bruno, Canada). Cell lines were maintained as monolayers at 37 °C in a humidified 5% CO_2_ atmosphere.

### Cytotoxicity assay

OVCAR-3, IGROV-1, SKOV-3, EFO-21 (5 × 10^3^) or A2780 cells (2.5 × 10^3^) were seeded in 96-well plates and allowed to attach overnight (37 °C, 5% CO_2_). Twenty-four hours after plating, cells were treated with a dose range of drugs (Carboplatin, Gefitinib, Crizotinib, Dasatinib or the equieffective combinations of Carboplatin + Crizotinib, Carboplatin + Dasatinib, Carboplatin + Gefitinib, Carboplatin + Crizotinib + Dasatinib, Carboplatin + Crizotinib + Gefitinib or Carboplatin + Dasatinib + Gefitinib). The equieffective combinations were defined by a ratio depending on the IC50 of each individual drug. After 72 h of treatment, cells were fixed in 50% trichloroacetic acid (TCA) for 2 h at 4 °C and washed four times under tap water. Cells were stained with sulforhodamine B (0.4%) overnight at room temperature [33]. Plates were then rinsed four times with 1% acetic acid and allowed to dry overnight. Stained cells were dissolved using 10 mM Tris-Base and the plates were read using a microplate reader ELx808 at 492 nm. The results were analyzed using GraphPad Prism 6.0 (GraphPadSoftware, Inc., San Diego, CA) to determine IC50 values. In one experiment, each condition was realized three times and each experiment was performed at least three times. The combination indices (CI) and the combination dot plots were determined with the CompuSyn 1.0 software.

### Annexin V-FITC/PI assay

OVCAR-3, IGROV-1, SKOV-3, A2780 and EFO-21 cells (7.5 × 10^4^) were seeded in 12-well plates and treated 24 h later with Dasatinib, Gefitinib (IC50 of each cell line after 72 h of treatment) or an equieffective combination of Dasatinib and Gefitinib (IC50 of each drug alone). Forty eight hours after treatment, cells were washed twice with fresh PBS, trypsinized (supernatants are kept) and stained with a FITC-Annexin V/PI apoptosis detection kit (Affymetrix, eBioscience) according to the manufacturer’s protocol. FITC-Annexin staining and PI incorporation are measured in cells with a FACS Canto II flow cytometer and analyzed with FACS Diva. Early apoptotic cells were defined as Annexin V-positive/PI-negative cells. Ten thousands events were counted for the first experiment and 20,000 events were counted for the second and third experiments (replicates). In one experiment, each condition was realized two times and each experiment was performed three times.

### Migration assay

Twenty four-well polycarbonate Transwell migration inserts (8.0 μm/6.5 mm, Corning Costar) were seeded with 10^5^ HOAC (OVCAR-3, IGROV-1, SKOV-3, A2780 and EFO-21) in 200 μL of serum-free RPMI in the upper compartment. After 4 h, 200 μL of serum-free RPMI was added in the upper compartment and 1 mL of complete RPMI was added in the lower compartment to trigger cell migration. Cells were treated in both lower and upper compartments with Dasatinib, Gefitinib (0.1 × IC50 of each cell line after 72 h of treatment) or an equieffective combination of Dasatinib and Gefitinib (0.1 × IC50 of each drug alone). Twenty four hours later, the medium was aspirated from the inserts and the latter were fixed with formaldehyde 3.7% during 20 min then washed three times with H_2_O. Cells were stained with crystal violet during 20 min and washed three times with H_2_O. Non-migrated cells were removed using a cotton swab then allowed to dry overnight. Ten pictures per condition were taken then migrated cells were counted using the Image J software. In one experiment, each condition was realized two times and each experiment was performed three times.

### Invasion assay

Fifty microliter of 6% Matrigel (Costar Corning) was added on the top of 24-well polycarbonate Transwell migration inserts (8.0 μm/6.5 mm, Corning Costar) at least 30 min at 37 °C for polymerization. The migration assay protocol was applied for the next steps. In one experiment, each condition was realized two times and each experiment was performed three times.

### Western blot

HOAC (10^6^) were harvested in 6-well plates and treated 2 h or 24 h with Dasatinib, Gefitinib (IC50 of each cell line after 72 h of treatment) or an equieffective combination of Dasatinib and Gefitinib (IC50 of each drug alone). After the treatment, cells were washed twice with cold PBS and scraped in cold lysis buffer 50 mM Tris-HCl pH 7.5; 150 mM NaCl; 1% Nonidet P-40, 1 mM EDTA; 5 mM NaF; 1 mM Na3VO4; protease inhibitor tablet (Roche Biochemicals, Laval, Canada). Lysates were kept on ice for 30 min and collected by centrifugation at 14,000 g for 15 min at 4 °C. The concentration of protein was determined using the Bio-Rad protein assay kit (Bio-Rad laboratories, Hercules, CA). Proteins (50 μg) were separated by SDS-PAGE on a 4–15% polyacrylamide precast gel (BioRad). Proteins were transferred on a PVDF membrane (previously activated with methanol). Membranes were saturated for 1 h in TBS (50 mM Tris, 150 mM NaCl)/0,1% Tween 20/5% milk and incubated overnight at 4 °C with phosphotyrosine antibodies directed against: EGFR (Tyr1068, D7A5, Rabbit, 1:4000, Cell Signaling), c-Src (Tyr416, D49G4, Rabbit, 1:1000, Cell Signaling), Erk (9101, Thr202/Tyr204, 1:1500, Rabbit, Cell Signaling) or Akt (587 F11, Ser473, 1:2000, Rabbit, Cell Signaling); or with antibodies directed against total proteins: EGFR (1005, Rabbit, 1:1000, Santa Cruz Biotechnology), c-Src (2108, Rabbit, 1:1000, Cell Signaling), Erk (L34F12, Mouse, 1:1200, Cell Signaling) or Akt (9272, Rabbit, 1:1000, Cell Signaling). Membranes were washed three times with TBS/0.1% Tween 20 (TT) then incubated for 1 h30 with a secondary antibody (according to the specie of the primary antibody) coupled with HRP (Horse Raddish Peroxydase). Membranes were washed three times with TT and a further two times with TBS. After incubation with antibodies, the membranes were stripped using the Restore Stripping buffer (Thermo Scientific, Rockford, IL, United States) and probed for beta-actin (Santa Cruz, CA, USA), as the loading control. Immunoblot bands were visualized using Pierce™ ECL Western Blotting Substrate (Life Technologies Inc., ON, Canada).

### Statistical analysis

For this entire study, statistical significance was reached by *p* < 0.05. In vitro group comparisons were made using the Mann-Whitney test or the student *t* test (independent values) for non-parametric data. Each experiment was performed at least three times with independent samples (biological replicates).

## Results

### Individual kinase inhibitors induce a moderate cell-specific sensitization of HOAC to carboplatin

**Fig. 1:**
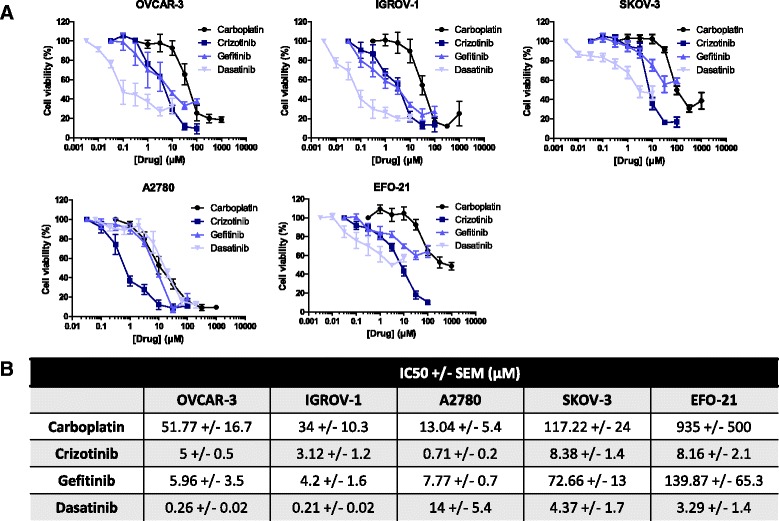
In vitro inhibition of HOAC viability by carboplatin or kinase inhibitors in monotherapy. HOACs were treated with a dose range of carboplatin, Crizotinib, Dasatinib or Gefitinib in monotherapy and showed cell-specific sensitivity or resistance. **a** 72 h after treatment, cell viability was determined by a colorimetric assay using SRB (Mean +/− SEM, n ≥ 3). **b** The IC50 of carboplatin or kinase inhibitors after 72 h of treatment were determined for each cell line (Mean +/− SEM, n ≥ 3)

We aimed to determine if inhibitors of Met, c-Src and EGFR, respectively Crizotinib, Dasatinib or Gefitinib, were able to sensitize HOAC to carboplatin. We decided to work on a panel of carboplatin-sensitive (OVCAR-3, IGROV-1, A2780; IC50 from 13 to 52 μM) or carboplatin-resistant (SKOV-3, EFO-21; IC50 from 120 to 935 μM) cell lines (Fig. 1a and b). Most of the tested cell lines showed a relative resistance to Crizotinib alone (IC50 from 3.12 to 8.38 μM) except for A2780 with a low IC50 of 0.71 μM. As for the carboplatin, OVCAR-3, IGROV-1 and A2780 cells were sensitive to Gefitinib alone (IC50 from 4.2 to 7.77 μM) whereas SKOV-3 and EFO-21 cells were more resistant (IC50 from 72.66 to 139.87 μM). Finally, OVCAR-3 and IGROV-1 cells were sensitive to a treatment with Crizotinib alone with sub-millimolar IC50 (from 0.21 to 0.26 μM) contrary to A2780, SKOV-3 and EFO-21 cells (IC50 from 3.29 to 4.37 μM).

**Fig. 2:**
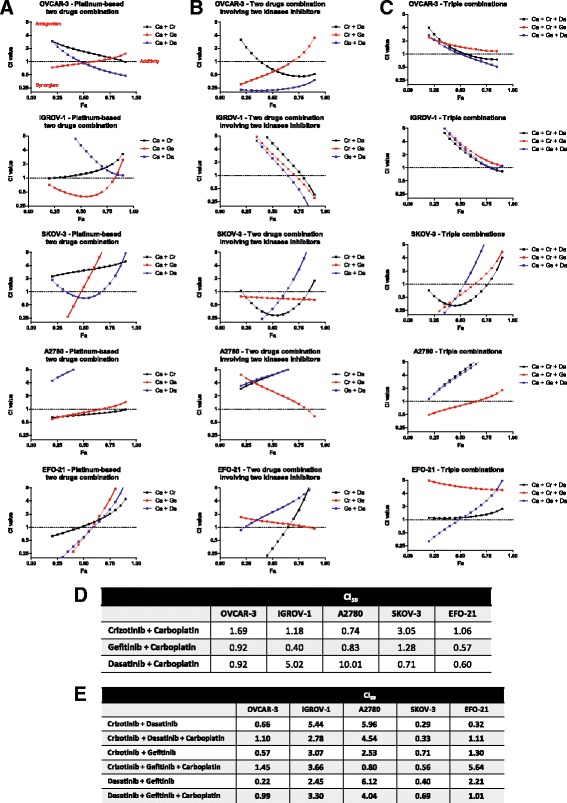
In vitro inhibition of HOAC viability by kinase inhibitors in tandem and/or in combination with carboplatin. Individual kinase inhibitors or two-kinase inhibitor combinations induce a cell-specific sensitization of HOAC to carboplatin. Combination index (CI)-fraction affected (Fa, corresponding to the fraction of cell viability inhibited) plot of HOACs treated with (**a**) a dose range of carboplatin (Ca) in combination with dose range of Crizotinib (Cr), Dasatinib (Da) or Gefitinib (Ge), based on a ratio of the IC50 of the two drugs, (**b**) kinase inhibitors in a tandem combination based on a ratio of the IC50 of the two drugs or (**c**) kinase inhibitors in tandem in combination with carboplatin. The *dotted line* represents the additivity while the synergism is considered below the line (CI < 1) and the antagonism above the line (CI > 1). **d** and **e** The combination index CI50 were determined for each drug combination

In order to test the efficacy of the combination between carboplatin and the previously tested kinase inhibitors, we realized equieffective combinations of these drugs using a ratio depending on the IC50 of each individual drug and each cell line. The combination index dot plots and isobolograms of these drugs were generated at all fractions affected (Fa) with the CompuSyn software (Fig. 2a and d, Additional files 1 and 2), based on the Chou and Talalay equations (synergy (CI < 1), antagonism (CI > 1) or additive effect (CI = 1 or close to 1)) [34]. The equieffective combination of carboplatin plus Crizotinib was antagonistic in OVCAR-3, IGROV-1 and SKOV-3 cells (CI > 1 for all Fa) but synergistic in A2780 cells at all Fa (CI < 1). In EFO-21 cells, carboplatin plus Crizotinib was synergistic for Fa lower than 50% but antagonistic above this value. Likewise, the combination of carboplatin with Gefitinib in OVCAR-3, SKOV-3, A2780 and EFO-21 cells was synergistic for low Fa (<0.5) and antagonistic for higher Fa. In IGROV-1, this combination appeared to be highly synergistic (CI < 1). Finally, the combination between carboplatin and Dasatinib was strongly antagonistic in IGROV-1 and A2780 cells (CI > 1 for all Fa). A sub-additive effect was observed for SKOV-3 cells (CI close to 1). In OVCAR-3 cells, the carboplatin and Dasatinib combination was synergistic for high Fa (>0.5) while in EFO-21, this combination was synergistic only at low Fa (<0.6). Synergy between Crizotinib, Dasatinib or Gefitinib in combination with carboplatin was cell-specific in HOAC.

### A two-kinase inhibitors combination induces a cell-specific sensitization of HOAC to carboplatin

To further study a possible platinum-sensitizer effect of kinase inhibitors on HOAC, we decided to combine them in tandem with the carboplatin in an equieffective fashion (Fig. 2b, c, d and e, Additional files 3 and 4). The combination of Crizotinib and Dasatinib was synergistic in OVCAR-3 (for Fa > 0.4), EFO-21 (for Fa < 0.7) and SKOV-3 cells while this combination strongly antagonistic in the other cell lines of our panel. However, the addition of carboplatin to this combination did not increase potency. Indeed, the combinations of carboplatin with kinase inhibitors were mostly antagonistic in all cell lines except for SKOV-3 cells. Likewise, the combination of Crizotinib and Gefitinib was additive in SKOV-3 and EFO-21 cells (CI ≈ 1) and antagonistic in IGROV-1 and A2780 cells. Only OVCAR-3 cells were sensitive to this combination, with a synergistic effect observed at low Fa (below 0.7). Again, the addition of carboplatin to these two kinase inhibitors led to antagonistic effect in OVCAR-3, IGROV-1 and EFO-21 cells compared to the Crizotinib and Gefitinib combination. However, the triple combination was synergistic in SKOV-3 and A2780 cells for low Fa (below 0.6). Finally, Dasatinib and Gefitinib combination exhibited a high potency with a synergy in OVCAR-3, IGROV-1 (for Fa > 0.6) and SKOV-3 cells (for Fa < 0.6). As for the combination of Gefitinib with Crizotinib, addition of carboplatin to Dasatinib and Gefitinib combination led to antagonistic effect.

These results indicate that the use of two-drug combinations induced cell-specific synergistic effects. Moreover, the addition of carboplatin with to kinase inhibitors tandems did not show a clear improvement of the overall cytotoxic effect.

Dasatinib and Gefitinib combination showed a synergy in all cell lines except for A2780 and EFO-21 cells where we observed an antagonistic effect. Thus, we decided to focus our further studies on this combination.

### The Dasatinib and Gefitinib synergic effect does not imply an apoptosis induction

**Fig. 3:**
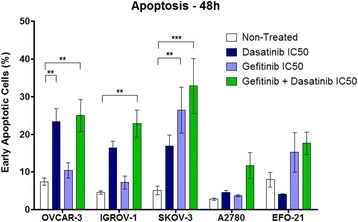
In vitro induction of apoptosis in HOACs by Dasatinib, Gefitinib or a combination of both drugs. The Dasatinib and Gefitinib synergic effect does not imply apoptosis induction. HOACs were treated with Dasatinib, Gefitinib (IC50 after 72 h of treatment for each cell line) or an equieffective combination of both treatments. The negative control corresponds to non-treated cells. Forty-eight hours after treatment, cells were stained with a FITC-Annexin V/PI apoptosis detection kit. Early apoptotic cells correspond to the Annexin V positive and PI negative population. (Mean +/− SEM, ** = *p* < 0.01, *** = *p* < 0.001 *n* = 3)

Dasatinib and Gefitinib combination presented a potent cytotoxic effect in OVCAR-3, IGROV-1 (Fa > 0.6) and SKOV-3 cells (Fa < 0.6). We aimed to decipher the underlined cell death mechanism behind this synergy. We then treated HOAC with each drug alone or equieffective combinations (each drug at its IC_50_) and studied the induction of apoptosis by flow cytometry (Fig. 3). The Dasatinib alone significantly increased the percentage of Annexin V-positive/PI-negative cells, defined as early apoptotic cells, compared to the control (non-treated cells) in OVCAR-3 (from 7.5 to 23%) whereas we observed no significant effect in other cell lines even though there was a weak induction of early apoptosis in IGROV-1 and SKOV-3 cells (from 4.5 and 5 to 16 and 17% respectively). The treatment with Gefitinib alone induced a significant increase in the percentage of early apoptotic SKOV-3 cells (from 5 to 26.5%) but no effect in the other cell lines except for EFO-21 with a weak apoptosis induction (from 8 to 15%). The equieffective combination of Dasatinib and Gefitinib induced a significant increase in the percentage of early apoptotic cells in OVCAR-3, IGROV-1 and SKOV-3 cells (from 7.5, 4.5 and 5 to 25, 23 and 33% respectively). This combination induced a weak but not significant increase of this percentage in EFO-21 cells (from 8 to 17.5%) and more surprisingly in A2780 cells (from 3 to 12%) where the Dasatinib and Gefitinib combination showed no cytotoxic synergy. However, despite the fact that tandem kinase inhibitor combination induced apoptosis in our HOAC, we observed no synergy nor superior effect compared with single drug alone. Likewise, the analysis of late apoptotic and necrotic cells (Annexin V-positive/PI-positive cells) did not reveal any synergy for the combination of Dasatinib and Gefitinib (Additional file 5). These results indicate that the Dasatinib and Gefitinib synergistic effect observed in some of our HOAC does not lead to an increased induction of apoptosis.

### The Dasatinib and Gefitinib combination suppresses HOAC migration and invasion

**Fig. 4:**
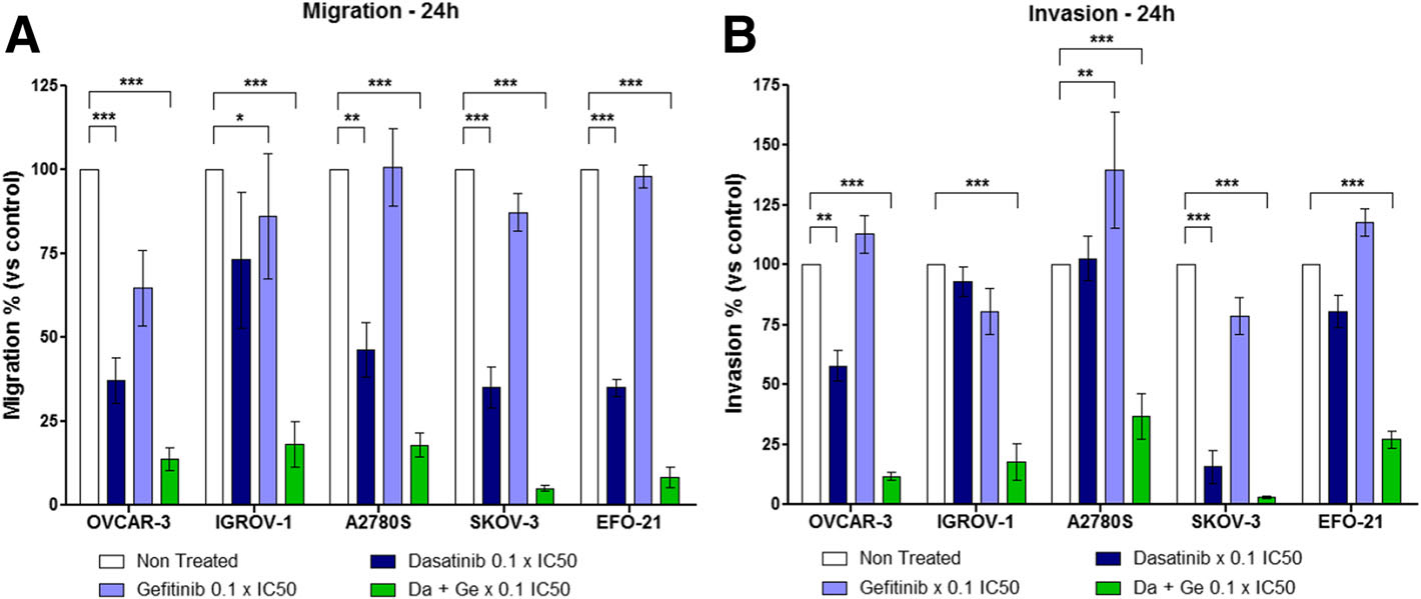
In vitro regulation of HOAC migration and invasion by Dasatinib, Gefitinib or a combination of both drugs. The Dasatinib and Gefitinib combination suppresses HOAC migration and invasion. HOAC were seeded in Transwell migration inserts in serum-free medium (**a**) or on a mix of matrigel matrix and serum-free medium (**b**). After 4 h, complete medium was added to the lower compartment and cells were treated with Dasatinib, Gefitinib (0.1 × IC50 of each cell line after 72 h of treatment) or an equieffective combination of Dasatinib and Gefitinib (Da + Ge) (0.1 × IC50 of each drug alone). Twenty-four hour later, migrating cells or invading cells were counted (Mean +/− SEM, ** = *p* < 0.01, *** = *p* < 0.001 n ≥ 3)

We then assessed the capacity of Dasatinib, Gefitinib or the combination of both drugs, to regulate HOAC migration through a polycarbonate insert. To avoid a too strong effect of kinase inhibitors, we treated HOAC during 24 h with a concentration of drugs of 0.1 × IC50 (Fig. 4a, Additional file 6A). Dasatinib alone inhibited from 25 to 70% of HOAC migration depending on the cell lines. On the contrary, Gefitinib alone presented a very weak effect with a maximum cell migration inhibition of 35% on OVCAR-3 cells and almost no effects on other cells lines. However, the combination of Dasatinib and Gefitinib suppressed most of HOAC migration (from 80 to 95% depending on cell lines) indicating a synergy of these two drugs.

We studied the effects of the Dasatinib and Gefitinib combination on the invasion of HOAC through a matrigel matrix. As for the migration assay, we treated HOAC during 24 h with a concentration of 0.1 × IC50 of Dasatinib, Gefitinib or a combination of both drugs (Fig. 4b, Additional file 6B). Dasatinib alone induced variable results on HOAC invasion with no effect on IGROV-1 and A2780 cells, an inhibition of 20 and 40% of cell invasion on EFO-21 and OVCAR-3 respectively, and a strong invasion inhibition on SKOV-3 (85%). As for cell migration, Gefitinib alone showed weak effects on HOAC invasion with an unexpected induction in OVCAR-3, EFO-21 and A2780 cells. Again, the combination of Dasatinib and Gefitinib suppressed most of HOAC invasion (from 65 to 95% depending on cell lines) indicating a synergy of these two drugs.

### The Dasatinib and Gefitinib combination inhibits EGFR, c-Src, Erk and Akt signaling

**Fig. 5:**
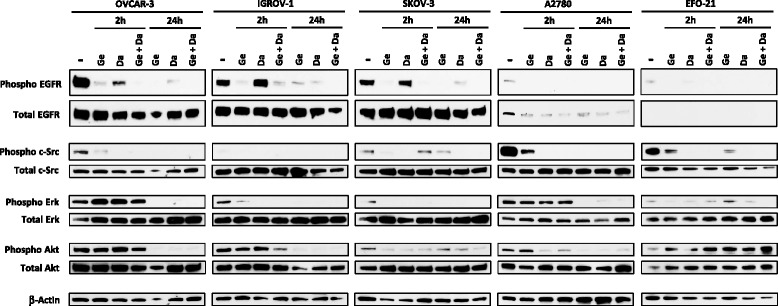
In vitro molecular effects of Dasatinib, Gefitinib or a combination of both drugs in HOAC signaling pathways. HOAC were treated 2 h or 24 h with Dasatinib (Da), Gefitinib (Ge) (0.1 × IC50 of each cell line after 72 h of treatment) or an equieffective combination of Dasatinib and Gefitinib (Da + Ge) (0.1 × IC50 of each drug alone). The EGFR, phospho-EGFR, c-Src, phospho-c-Src, ERK, phospho-ERK, Akt, phospho-Akt (p-Akt) and β-actin levels were determined by western blot using specific antibodies. Immunocomplexes were visualized by autoradiography

We finally wanted to decipher the effects of the Dasatinib and Gefitinib combination on HOAC cell proliferation and survival signaling pathways. We focused our study on the MAP Kinase pathway, mainly responsible for cell proliferation, and its members: EGFR, an upstream receptor directly targeted by the Gefitinib, and Erk, the final downstream protein of this pathway (Fig. 5). We also observed c-Src, an intracellular tyrosine kinase targeted by the Dasatinib, and Akt, a key member of the PI3K/Akt survival pathway that can be regulated by both EGFR and c-Src.

We first noticed differences in the basal EGFR, Erk, c-Src and Akt phosphorylation between our HOAC. Indeed, EGFR is strongly phosphorylated in OVCAR-3, IGROV-1 and SKOV-3 cells whereas its phosphorylation is low in A2780 and EFO-21 cells. In basal conditions, Erk is phosphorylated in all cell lines with a low activation in EFO-21 cells, c-Src is phosphorylated in all cell lines except for IGROV-1 and Akt is phosphorylated in all cell lines. Gefitinib alone totally inhibited EGFR phosphorylation but also partially inhibited c-Src in all cell lines with a maximum effect after 24 h of treatment. Erk phosphorylation was inhibited by Gefitinib alone after 2 h in IGROV-1, SKOV-3 and EFO-21 cells but only after 24 h in OVCAR-3 and A2780 cells. Similarly, Akt phosphorylation was directly inhibited after 2 h of treatment with Gefitinib alone in SKOV-3 cells but only after 24 h in the other cell lines except for EFO-21 where the treatment had no effect. The treatment with Dasatinib alone during 2 h had no effect on the EGFR phosphorylation in HOAC except a slight inhibition in A2780 and EFO-21 where its basal level was already very low. However, after 24 h of Dasatinib treatment, we observed a nearly complete inhibition of EGFR phosphorylation in all HOAC. c-Src phosphorylation was totally inhibited in all cell lines from 2 h of this treatment. As for the Gefitinib treatment, Dasatinib alone inhibited Erk phosphorylation after 2 h in IGROV-1, SKOV-3 and EFO-21 cells but only after 24 h in OVCAR-3 and A2780 cells. Akt phosphorylation was directly inhibited after 2 h of this treatment in SKOV-3 and A2780 cells, 24 h in the OVCAR-3 and IGROV-1 cells, and was not altered in EFO-21 cells. The combination of Dasatinib and Gefitinib strongly inhibited EGFR and c-Src phosphorylation in all cell lines with a maximum effect after 24 h of treatment. As for the single treatments alone, the combination inhibited Erk phosphorylation after 2 h in IGROV-1, SKOV-3 and EFO-21 cells but only after 24 h in OVCAR-3 and A2780 cells. Likewise, the Akt phosphorylation was inhibited by the combination of Dasatinib and Gefitinib directly after 2 h in SKOV-3 and A2780 and after 24 h in OVCAR-3 and IGROV-1. This combination was not able to modify the level of phosphorylated Akt in EFO-21 cells.

Finally, in all the tested HOAC (with the exception of EFO-21 for Akt phoshoprylation), we had the confirmation that the combination of Dasatinib and Gefitinib was able to inhibit the phosphorylation of their targets, EGFR and c-Src, as well as the phosphorylation of the downstream protein Erk and the Akt survival protein. This tandem combination induced higher effects that each drug alone and further proves the synergy of this association. Moreover, no compensatory activation of the studied signaling pathways was observed after the treatment of HOAC with single drugs or the tandem combination of Dasatinib and Gefitinib.

## Discussion

Since the introduction of carboplatin 35 years ago as the reference treatment for advanced stage ovarian cancers, few new therapies have increased the overall survival of patients. Bevacizumab, a monoclonal antibody targeting vascular endothelial growth factor (VEGF), in combination with conventional chemotherapies increased disease-free survival without modifying the overall survival of patients and olaparib, a Poly ADP-ribose Polymerase (PARP) inhibitor showed promising results in combination with carboplatin in platinum-sensitive patients [35, 36]. However, for platinum-resistant patients, new agents are urgently needed to sensitize ovarian tumors to carboplatin. EGFR, c-Src and Met activation being associated with induction of DNA repair, we sought to study their clinical inhibitors combination with carboplatin [4–11].

Our equieffective combinations of kinase inhibitors with carboplatin and/or another kinase inhibitor showed variable results with ovarian cancer cell-specific synergies (Figs. 1 and 2). As an example, in OVCAR-3 cells, all the tandem combinations of kinase inhibitors showed synergistic effects (CI_50_ < 0.8, Fig. 2e) whereas the addition of carboplatin always increased the CI_50_ and thus decrease the synergistic potential of the treatment. On the contrary, in SKOV-3 cells, carboplatin was synergistic with all our kinase inhibitors alone or in a tandem combination (except for high Fa). These results indicate the crucial need of biomarkers to select which ovarian cancer could be sensitized by the treatment. Huang et al. [37] have developed a six-gene model to predict Dasatinib efficacy in primary breast, lung and ovarian cancer patients. They indicated that most responsive breast tumors had the highest expression of Keratin 5/7 (KRT5/KRT17) and the lowest expression of endogen receptor (ER), progesterone receptor (PR) or human epidermal growth factor receptor-2 (HER2). Another study showed in non-small cell lung cancer patients that a positive phospho-Erk activity might be correlated with a poor response to Gefitinib while high phospho-Akt combined with a negative phospho-Erk, or EGFR mutations associated with expression of HER2/3 could predict efficiency of this drug [38, 39]. Nevertheless, our results show promising synergistic effects of Dasatinib/carboplatin and Gefitinib/carboplatin combinations on carboplatin-resistant cell lines SKOV-3 and EFO-21 cell lines, raising hopes for carboplatin-resistant ovarian cancer patients.

The current study was focused on Gefitinib and Dasatinib combination because of its synergistic potential in some of our cell lines. This combination may well represent a promising strategy for sensitizing ovarian tumours to platinum-based therapies [40]. Despite the capacity of each individual drug to induce a cell line-specific apoptosis, the combination of Gefitinib and Dasatinib did not significantly increase the percentage of early apoptotic cells (Fig. 3) [41–45]. These results indicate that the cytotoxic synergy previously observed between Gefitinib and Dasatinib does not imply the induction of apoptosis but could rather be due to a proliferation inhibition and/or the activation of another cell death pathway. Indeed, Gefitinib is able to trigger apoptosis and to inhibit proliferation in esophageal squamous cell carcinoma cell lines [46]. Likewise, Dasatinib exhibited an antiproliferative activity but did not trigger apoptosis in neuroblastoma and Ewing sarcoma cells whereas the same treatment induced autophagy in HEY and SKOV-3 ovarian cancer cells [47, 48].

As observed in other tumor models, Dasatinib and Gefitinib inhibited migration and invasion in some of our HOAC [49–52]. The combination of small concentrations (0.1 × IC50) of Dasatinib and Gefitinib highly inhibited HOAC migration and invasion compared to single treatments, even in A2780 and EFO-21 cells where we did not observe any cytotoxic synergy for this treatment (Fig. 4). Surprisingly, the use of Gefitinib alone enhanced the invasion of OVCAR-3, A2780 and EFO-21 cells strengthening the need to use Dasatinib and Gefitinib in combination. These data represent promising results and indicate a potential capacity of this kinase inhibitors combination to prevent ovarian cancer metastasis and dissemination, even for tumors showing no anti-tumor synergy compared to each single drug alone (e.g. A2780 cells).

Our HOAC presented different levels of basal expression and activation of EGFR and c-Src (Fig. 5). Ono et al. [53] already showed that the expression of EGFR and HER2 were not correlated with Gefitinib sensitivity in non-small cell lung cancer cells. Always in lung cancer cells, Dasatinib sensitivity was not correlated with c-Src level but could be associated with the presence of EGFR-activating mutations [54, 55]. We studied the molecular effects of Dasatinib, Gefitinib or a combination of both drugs on key signaling pathways implied in cell proliferation and survival. Single treatments, at the tested concentrations and with the exception of EFO-21 cells, did not activate any compensatory signaling on the observed proteins despite the numerous compensatory loops described in the literature [56–58]. Surprisingly, the EFO-21 cell line was the only one where the Dasatinib and Gefitinib combination triggered an increased Akt phosphorylation. Perhaps, in this cell line, downregulation of Akt phosphorylation resulting from EGFR inhibition by Gefitinib, could lead to that of its effector mammalian target of rapamycin (mTOR), which could trigger a positive feedback responsible for a reactivation of Akt [59]. This same feedback mechanism could explain the moderate inhibition of c-Src phosphorylation observed in SKOV-3 cells after 2 h of Dasatinib and Gefitinib treatment while compared with drug alone.

We showed very promising in vitro cytotoxic, anti-migratory and anti-invasive properties of a Gefitinib and Dasatinib combination in ovarian cancer cells that now requires validation in vivo. Intraperitoneal xenograft of HOAC in Nude mice could constitute the good model for studying the effects of this combination on peritoneal carcinomatosis and matching with the human disease, as realized in several studies [60–62]. Demonstrating the efficacy of combining two kinase inhibitors to maximize the anti-tumor effects of the drugs will be the key to offer new strategies for advanced stage ovarian cancer patients.

## Conclusions

Crizotinib, Dasatinib or Gefitinib, alone, in tandem and/or in combination with carboplatin showed synergistic interactions in a cell-specific manner. The study suggests that specific biomarkers need to be identified in order to determine which tumour types can benefit from the latter combinations.. The combination of Dasatinib and Gefitinib was efficient and synergistic in OVCAR-3, IGROV-1 (for high Fa) and SKOV-3 cells but did not induce more cell death by apoptosis. This combination suppressed cell migration, invasion and the activation of EGFR, Erk, c-Src and Akt in all HOAC compared to the single treatment. Dasatinib and Gefitinib combination thus represents a promising new therapeutic modality to be evaluated in advanced stage ovarian cancer patients.

## Additional files

Additional file 1: In vitro inhibition of HOAC viability by carboplatin alone, or in combination with kinase inhibitors. HOACs were treated with a dose range of carboplatin alone or in combination with dose range of Crizotinib, Dasatinib or Gefitinib, based on a ratio of the IC50 of both drugs. Seventy-two hours after treatment, cell viability was determined by a colorimetric assay using SRB. The negative control (no treatment) of each condition corresponds to the 100% cell viability (Mean +/− SEM, n ≥ 3). (PDF 61 kb)

Additional file 2: In vitro inhibition of HOAC viability by carboplatin in combination with kinase inhibitors. HOACs were treated with a dose range of carboplatin (Ca) in combination with dose range of Crizotinib (Cr), Dasatinib (Da) or Gefitinib (Ge) based on a ratio of the IC50 of the two drugs. The IC50 of each drug are plotted on the axes and the circle represents the concentrations of each drug resulting in 50% of cell viability inhibition (Fa = 0.5). The solid line represents the additive effect. A synergistic combination is plotted on the left of the solid line while an antagonistic combination is plotted on the right. Isobolograms were generated with the CompuSyn 1.0 software. (PDF 39 kb)

Additional file 3: In vitro inhibition of HOAC viability by carboplatin alone, or in combination with two kinase inhibitors. HOACs were treated with a dose range of carboplatin alone or in combination with dose range of Crizotinib + Dasatinib, Crizotinib + Gefitinib, or Dasatinib + Gefitinib, based on a ratio of the IC50 of the three drugs. Seventy-two hours after treatment, cell viability was determined by a colorimetric assay using SRB. The negative control (no treatment) of each condition corresponds to the 100% cell viability (Mean +/− SEM, n ≥ 3). (PDF 75 kb)

Additional file 4: In vitro inhibition of HOAC viability kinase inhibitors in tandem. HOACs were treated with a dose range of Crizotinib (Cr), Dasatinib (Da) or Gefitinib (Ge) in tandem, based on a ratio of the IC50 of the two drugs. The IC50 of each drug are plotted on the axes and the circle represents the concentrations of each drug resulting in 50% of cell viability inhibition (Fa = 0.5). The solid line represents the additive effect. A synergistic combination is plotted on the left of the solid line while an antagonistic combination is plotted on the right. Isobolograms were generated with the CompuSyn 1.0 software. (PDF 39 kb)

Additional file 5: In vitro induction of late apoptosis and necrosis in HOACs by Dasatinib, Gefitinib or a combination of both drugs. HOACs were treated with Dasatinib, Gefitinib (IC50 after 72 h of treatment for each cell line) or an equieffective combination of both treatments. The negative control corresponds to non-treated cells 48 h after treatment, cells were stained with a FITC-Annexin V/PI apoptosis detection kit. FITC-Annexin staining and PI incorporation were measured in cells with a FACS Canto II flow cytometer and analyzed with FACS Diva. Late apoptotic and necrotic cells correspond to the Annexin V positive and PI positive population. (Mean +/− SEM, ** = *p* < 0.01, *** = *p* < 0.001 *n* = 3). (PDF 33 kb)

Additional file 6: In vitro regulation of HOAC migration and invasion by Dasatinib, Gefitinib or a combination of both drugs. HOAC were seeded in polycarbonate Transwell migration inserts in serum-free medium (A) or on a mix of matrigel matrix and serum-free medium (B). After 4 h, complete medium was added to the lower compartment and cells were treated with Dasatinib, Gefitinib (0.1 × IC50 of each cell line after 72 h of treatment) or an equieffective combination of Dasatinib and Gefitinib (Da + Ge) (0.1 × IC50 of each drug alone). Twenty-four hours later, cells were stained with crystal violet, 10 pictures per condition were taken. Representative pictures of n ≥ 3 replicates are represented. (PDF 1381 kb)

## Abbreviations

c-Src: cellular Src kinase
EGFR: Epidermal growth factor receptor
ER: Endogen receptor
Fa: Fraction affected
HER2: Human epidermal growth factor receptor-2
HGF: Hepatocyte Growth Factor
HOAC: Human ovarian adenocarcinoma cell
KRT: Keratin
Met: Hepatocyte growth factor receptor
mTOR: Mammalian target of rapamycin
PARP: Poly ADP-ribose Polymerase
PI3K: Phosphatidylinositol 3-kinase
PR: Progesterone receptor
SH2: Src-Homoloy-2
VEGF: Vascular endothelial growth factor
