# Supplementary figures and images for: Dasatinib + Gefitinib, a non platinum-based combination with enhanced growth inhibitory, anti-migratory and anti-invasive potency against human ovarian cancer cells
Source: J Ovarian Res. 2017 Apr 26;10:31. doi: 10.1186/s13048-017-0319-2 (PMC5405511; doi:10.1186/s13048-017-0319-2)

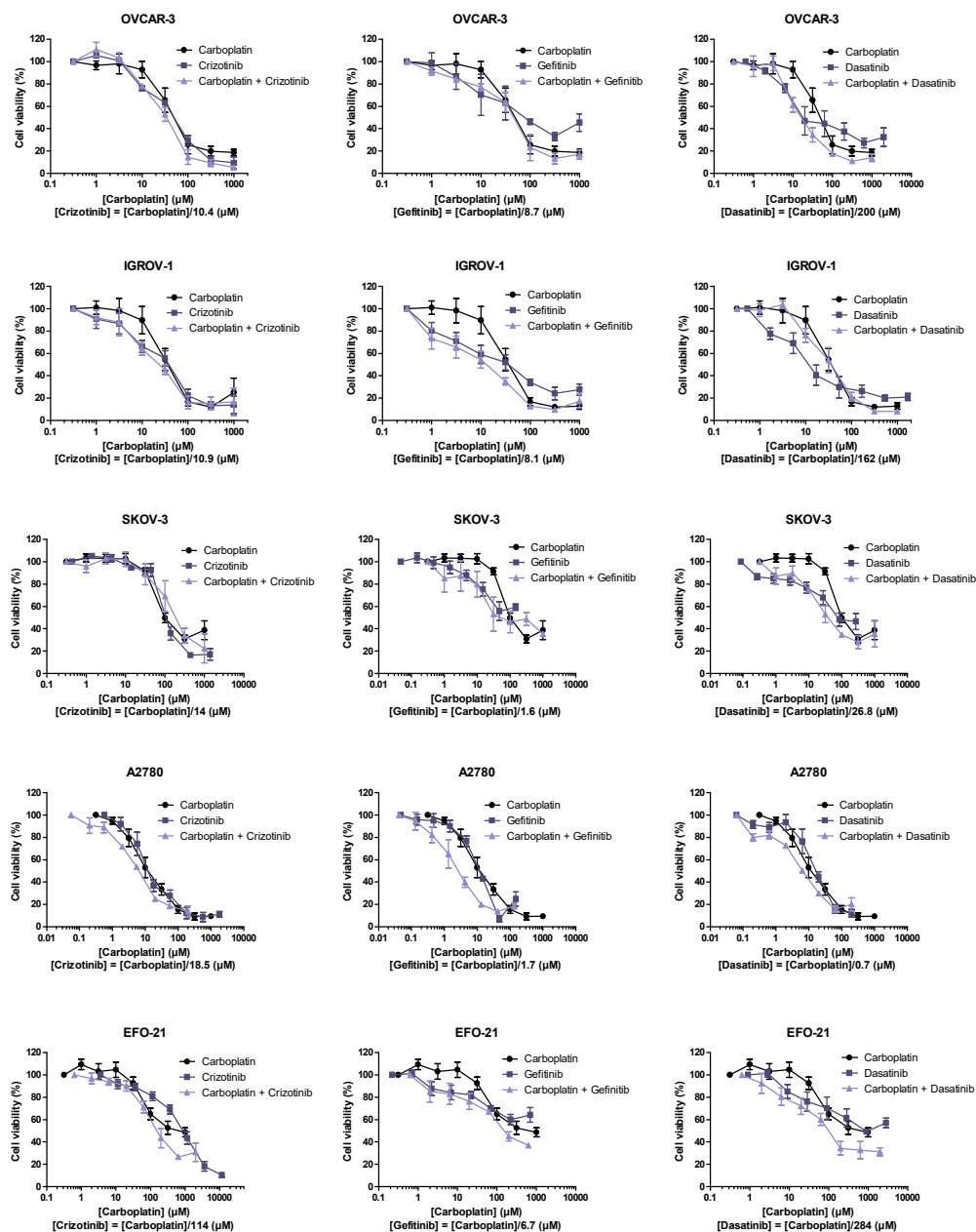

Additional file 1

Supplement: Supplementary file 1 — In vitro inhibition of HOAC viability by carboplatin alone, or in combination with kinase inhibitors. HOACs were treated with a dose range of carboplatin alone or in combination with dose range of Crizotinib, Dasatinib or Gefitinib, based on a ratio of the IC50 of both drugs. Seventy-two hours after treatment, cell viability was determined by a colorimetric assay using SRB. The negative control (no treatment) of each condition corresponds to the 100% cell viability (Mean +/− SEM, n ≥ 3). (PDF 61 kb) [file 13048_2017_319_MOESM1_ESM.pdf]

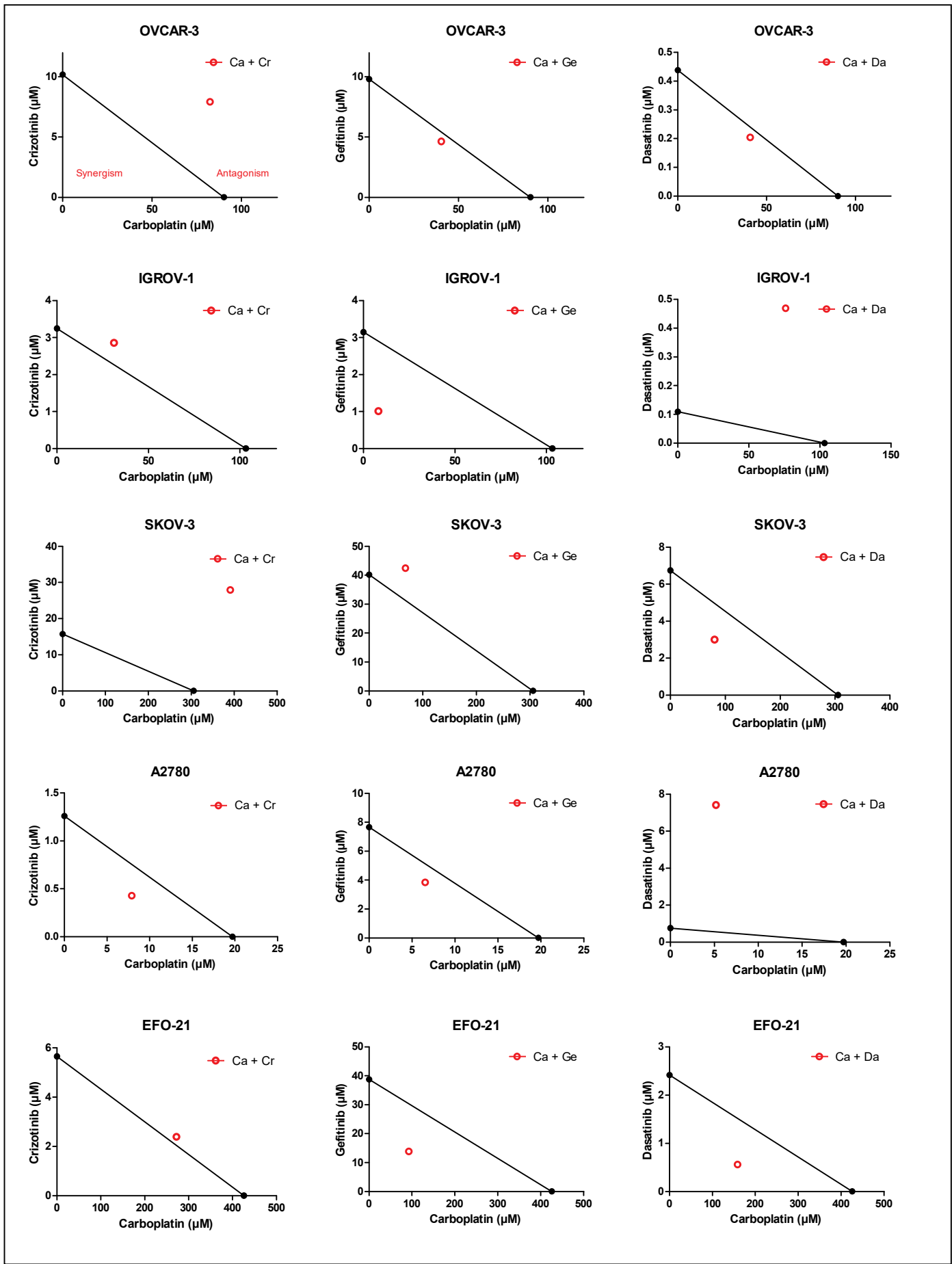

Additional file 2

Supplement: Supplementary file 2 — In vitro inhibition of HOAC viability by carboplatin in combination with kinase inhibitors. HOACs were treated with a dose range of carboplatin (Ca) in combination with dose range of Crizotinib (Cr), Dasatinib (Da) or Gefitinib (Ge) based on a ratio of the IC50 of the two drugs. The IC50 of each drug are plotted on the axes and the circle represents the concentrations of each drug resulting in 50% of cell viability inhibition (Fa = 0.5). The solid line represents the additive effect. A synergistic combination is plotted on the left of the solid line while an antagonistic combination is plotted on the right. Isobolograms were generated with the CompuSyn 1.0 software. (PDF 39 kb) [file 13048_2017_319_MOESM2_ESM.pdf]

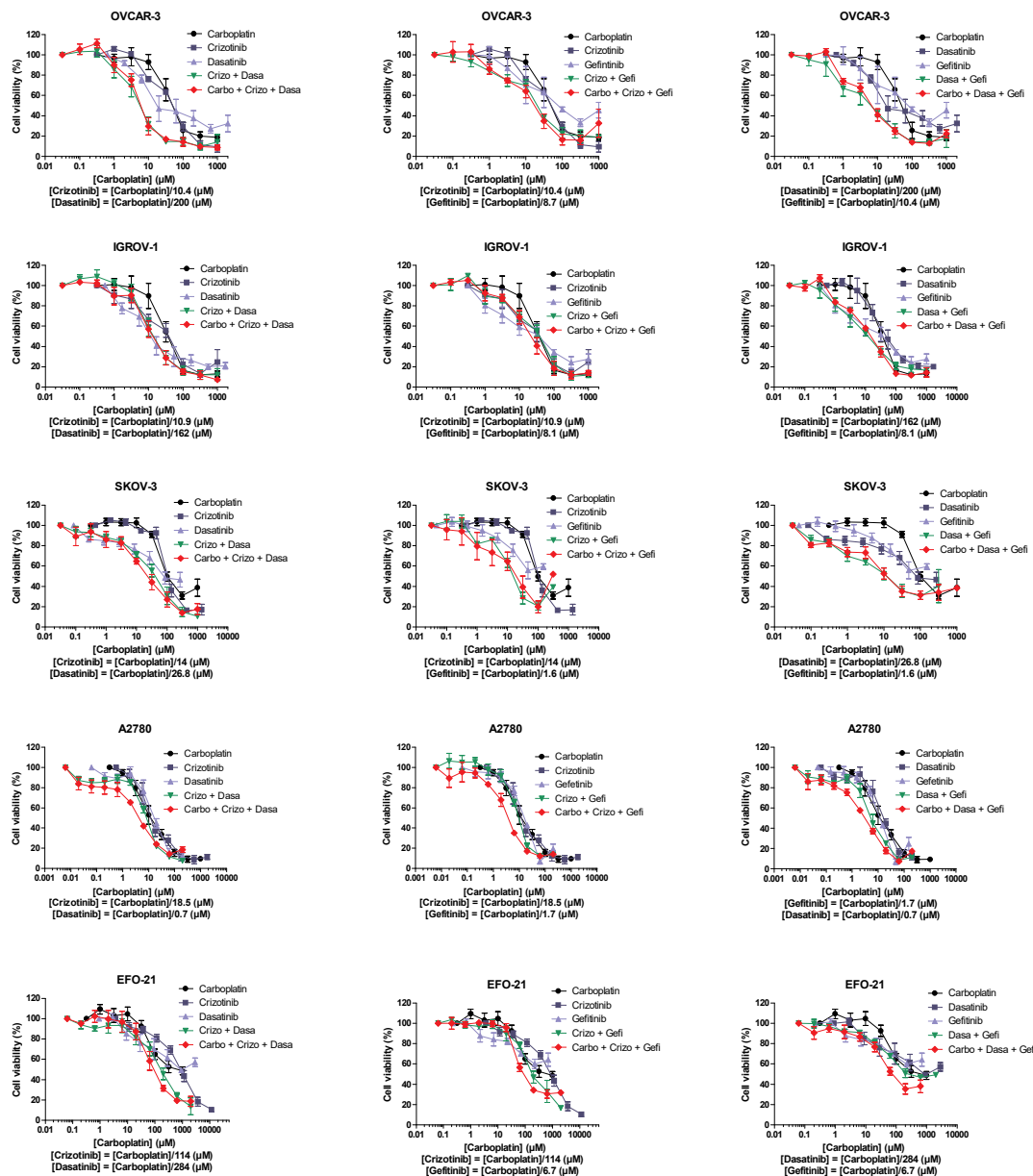

## Additional file 3

Supplement: Supplementary file 3 — In vitro inhibition of HOAC viability by carboplatin alone, or in combination with two kinase inhibitors. HOACs were treated with a dose range of carboplatin alone or in combination with dose range of Crizotinib + Dasatinib, Crizotinib + Gefitinib, or Dasatinib + Gefitinib, based on a ratio of the IC50 of the three drugs. Seventy-two hours after treatment, cell viability was determined by a colorimetric assay using SRB. The negative control (no treatment) of each condition corresponds to the 100% cell viability (Mean +/− SEM, n ≥ 3). (PDF 75 kb) [file 13048_2017_319_MOESM3_ESM.pdf]

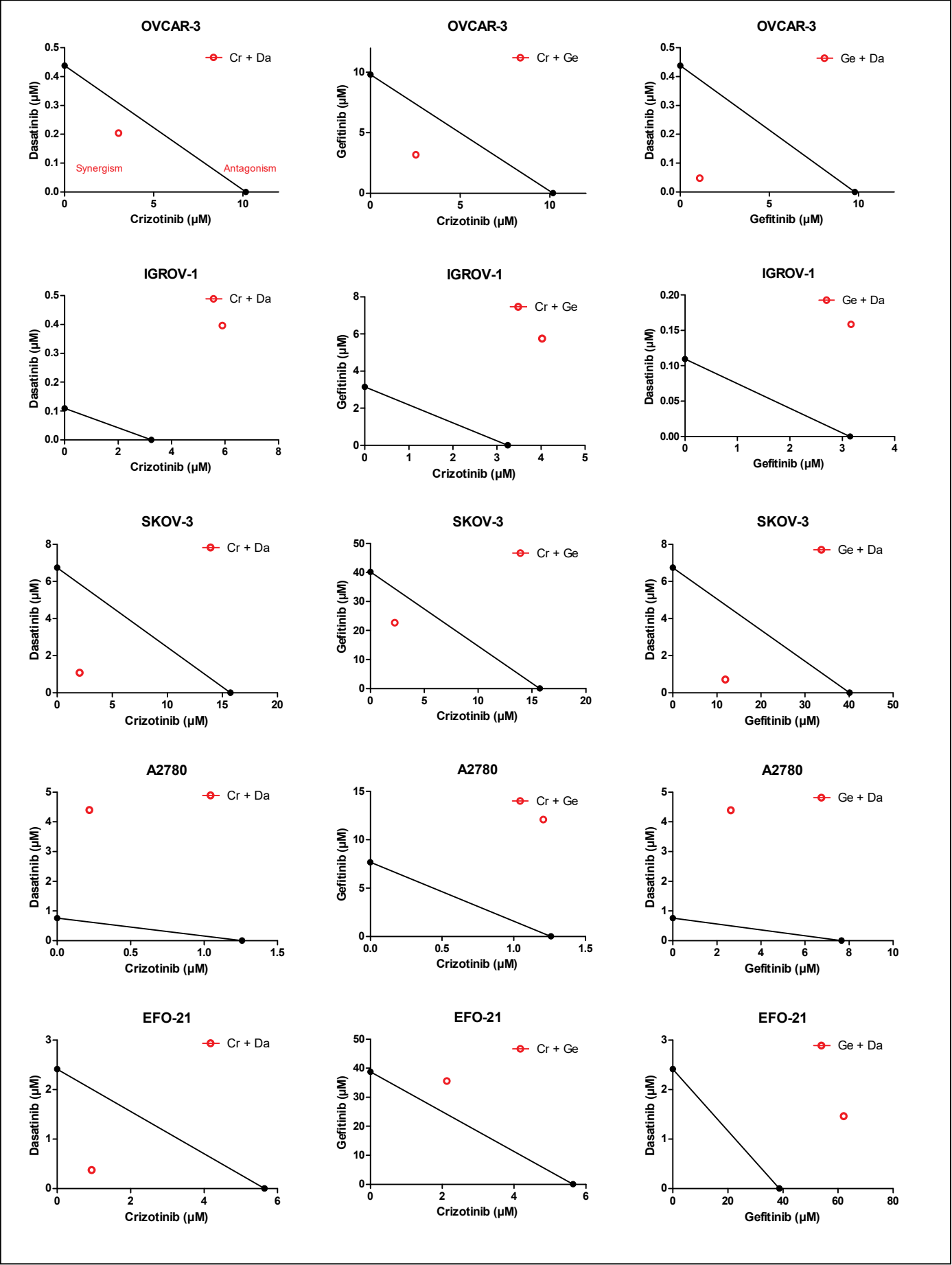

Additional file 4

Supplement: Supplementary file 4 — In vitro inhibition of HOAC viability kinase inhibitors in tandem. HOACs were treated with a dose range of Crizotinib (Cr), Dasatinib (Da) or Gefitinib (Ge) in tandem, based on a ratio of the IC50 of the two drugs. The IC50 of each drug are plotted on the axes and the circle represents the concentrations of each drug resulting in 50% of cell viability inhibition (Fa = 0.5). The solid line represents the additive effect. A synergistic combination is plotted on the left of the solid line while an antagonistic combination is plotted on the right. Isobolograms were generated with the CompuSyn 1.0 software. (PDF 39 kb) [file 13048_2017_319_MOESM4_ESM.pdf]

Late Apoptosis and Necrosis - 48h

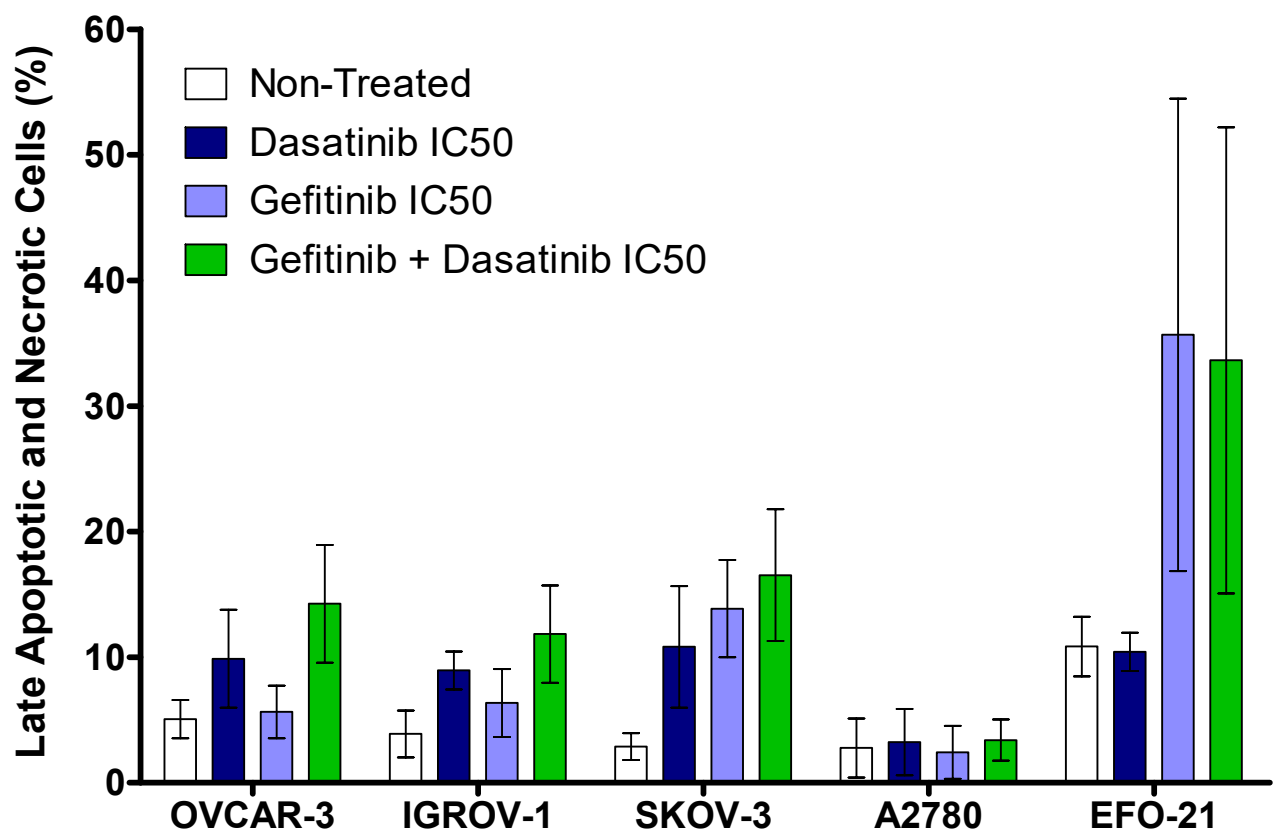

Additional file 5

Supplement: Supplementary file 5 — In vitro induction of late apoptosis and necrosis in HOACs by Dasatinib, Gefitinib or a combination of both drugs. HOACs were treated with Dasatinib, Gefitinib (IC50 after 72 h of treatment for each cell line) or an equieffective combination of both treatments. The negative control corresponds to non-treated cells 48 h after treatment, cells were stained with a FITC-Annexin V/PI apoptosis detection kit. FITC-Annexin staining and PI incorporation were measured in cells with a FACS Canto II flow cytometer and analyzed with FACS Diva. Late apoptotic and necrotic cells correspond to the Annexin V positive and PI positive population. (Mean +/− SEM, ** = p < 0.01, *** = p < 0.001 n = 3). (PDF 33 kb) [file 13048_2017_319_MOESM5_ESM.pdf]

## A Migration - 24h

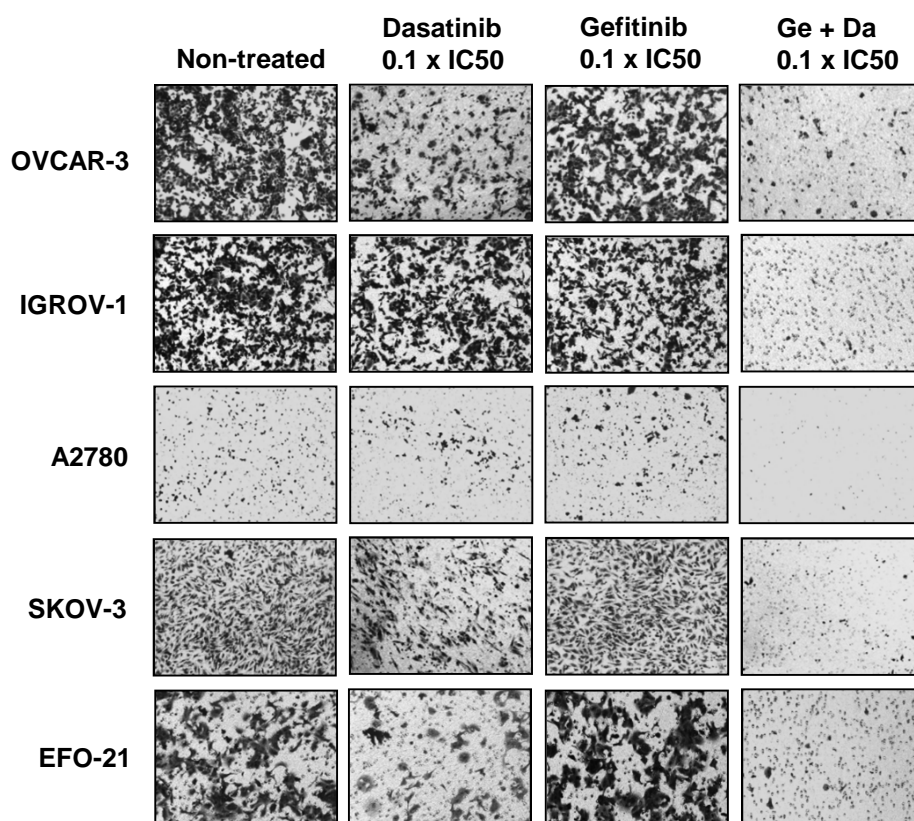

## B Invasion 24h

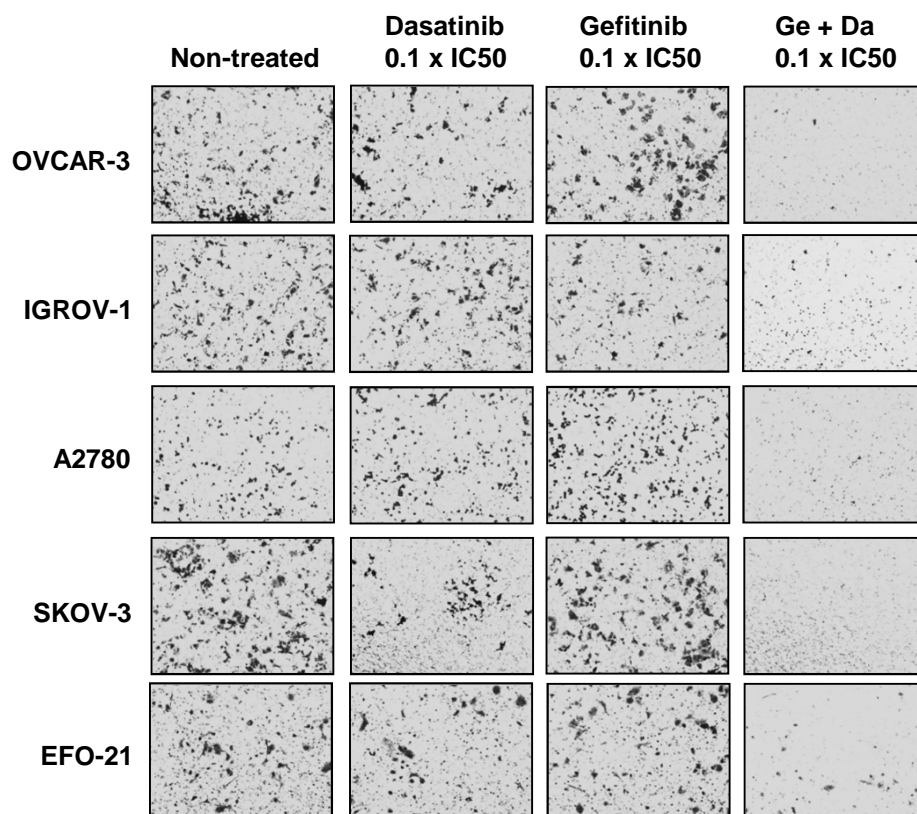

Additional file 6

Supplement: Supplementary file 6 — In vitro regulation of HOAC migration and invasion by Dasatinib, Gefitinib or a combination of both drugs. HOAC were seeded in polycarbonate Transwell migration inserts in serum-free medium (A) or on a mix of matrigel matrix and serum-free medium (B). After 4 h, complete medium was added to the lower compartment and cells were treated with Dasatinib, Gefitinib (0.1 × IC50 of each cell line after 72 h of treatment) or an equieffective combination of Dasatinib and Gefitinib (Da + Ge) (0.1 × IC50 of each drug alone). Twenty-four hours later, cells were stained with crystal violet, 10 pictures per condition were taken. Representative pictures of n ≥ 3 replicates are represented. (PDF 1381 kb) [file 13048_2017_319_MOESM6_ESM.pdf]
